# RESILIENCY PHENOTYPES FOLLOWING HIP FRACTURE IN OLDER ADULTS

**DOI:** 10.1093/geroni/igz038.3031

**Published:** 2019-11-08

**Authors:** Cathleen Colon-Emeric, Heather E Whitson, Carl Pieper, Richard Sloane, Denise Orwig, Kim Huffman, Daniel C Parker, Jay S Magaziner

**Affiliations:** 1 Duke University Medical Center, Durham, North Carolina, United States; 2 University of Maryland, Baltimore, Maryland, United States; 3 University of Maryland Baltimore School of Medicine, Baltimore, Maryland, United States

## Abstract

Defining common patterns of recovery after an acute health stressor (resiliency phenotypes) has clinical and research implications. We examined groups of patients with similar recovery patterns across 10 outcomes following hip fracture to determine the most important predictors of resiliency group membership. This study is a secondary analysis of three prospective cohort studies. Participants, community-dwelling adults aged >65 with recent surgical repair of a hip fracture (n=541), were recruited from eight hospitals near Baltimore and followed for up to one year. Self-reported function and activity measures were collected using validated scales at baseline, 2, 6, and 12 months. Physical performance tests were administered at all follow-up visits. Stressor characteristics, co-morbidities, psychosocial and environmental factors were collected at baseline, and latent class profile analysis was used to identify resiliency phenotypes and logistic regression models to identify associated factors. Three resiliency phenotypes had similar patterns across the 10 outcome measures and were defined as “high resilience” (n=163, 30.1%), “medium resilience” (n=242, 44.7%), and “low resilience” (n=136, 25.2%). Recovery trajectories for outcome measures were plotted for each resiliency group. Self-reported pre-fracture function was by far the strongest predictor of resilience group membership (AUC 0.84). Demographic factors, co-morbidities, stressor characteristics, environmental factors, and psychosocial characteristics were less predictive, but several factors remained significant in a fully adjusted model (AUC 0.88). These three resiliency phenotypes have immediate utility for clinical decision-making. They can be measured in future studies with a more parsimonious set of variables, and may prove useful for understanding mediators of physical resilience.

